# Refractory angina pectoris: a 20-year (2003–2022) bibliometric analysis

**DOI:** 10.3389/fcvm.2023.1228201

**Published:** 2023-08-24

**Authors:** Yunru Chen, Yaru Ge, Tiantian Chao, Na Huan, Wenjie Liu, Guojie Chu, Chenglong Wang

**Affiliations:** Center for Cardiovascular Disease, Xiyuan Hospital, China Academy of Chinese Medical Sciences, Beijing, China

**Keywords:** refractory angina, bibliometric analysis, cardiovascular diseases, citespace, knowledge mapping analysis

## Abstract

**Background:**

The increasing number of patients with refractory angina pectoris, combined with the aging population and improved survival rates among coronary heart disease patients, presents a significant challenge in contemporary cardiovascular medicine. The treatment of refractory angina has been an ongoing area of exploration, yet a comprehensive analysis of the existing literature on this topic is currently lacking. Therefore, this study aims to provide the first bibliometric analysis of publications related to refractory angina.

**Methods:**

A systematic search was conducted in the Web of Science database to identify articles related to refractory angina published between 2003 and 2022. The inclusion criteria were limited to articles and reviews written in English. CiteSpace software was utilized to conduct a collaborative network analysis of countries/regions, institutions and authors, co-occurrence analysis of keywords, and co-citation analysis of authors and references.

**Results:**

A total of 1,386 publications were identified, with an annual publication volume exhibiting fluctuation over time. American and European countries and institutions demonstrated a leading position in terms of research output. Henry TD emerged as the most prolific researcher in the field, while Mannheimer C received the highest number of citations. The primary research hotspot within this field focused on the treatment of refractory angina, with recent emphasis on emerging treatments such as stem cell therapy and the coronary sinus reducer. A significant number of clinical trials have been conducted, with a continuous focus on patient benefits, quality of life, and survival prognosis.

**Conclusion:**

Significant progress has been made in the field of refractory angina pectoris in recent years. Novel treatment methods, including spinal cord stimulation, enhanced external counterpulsation, stem cell therapy, and the coronary sinus reducer, hold promising therapeutic prospects. However, further high-quality evidence-based research is essential to support these emerging interventions. Additionally, the development of comprehensive evidence-based guidelines for refractory angina treatment is crucial. Such guidelines would provide clinicians with a framework to navigate the complexities of treatment choices and optimize patient care in this challenging condition.

## 1. Introduction

The 2019 ESC Guidelines for the Diagnosis and Management of Chronic Coronary Syndromes present a clear definition of refractory angina, a condition characterized by persistent symptoms lasting beyond three months. In the presence of obstructive coronary artery disease (CAD), refractory angina is caused by reversible ischemia. These distressing symptoms prove resistant to control even with the escalation of medical therapy using second- and third-line pharmacological agents, as well as interventions like bypass grafting, stenting, or percutaneous coronary intervention (PCI) of chronic total coronary occlusion (1). Unfortunately, refractory angina affects a significant portion, ranging from 5% to 15%, of individuals living with angina pectoris (2, 3). As a consequence, it not only diminishes their quality of life but also raises the risk of mortality (4).

To gain further insights into the field of refractory angina, this study adopted CiteSpace software and performed a bibliometric analysis of research accomplishments over the past two decades. Leveraging the comprehensive Web of Science (WOS) database, the analysis aimed to identify key trends, renowned researchers, main research countries and institutions, as well as the emerging research hotspots in this domain. By delving into the collective knowledge generated by the scientific community, this investigation sought to shed light on the advancements made in understanding and addressing refractory angina.

## 2. Materials and methods

### 2.1. Data source and filtering

To conduct this research, the data were sourced from the science citation index expanded (SCI-E) database on the esteemed WOS platform. Recognized as a highly reputable citation database for natural sciences, WOS provides an accurate reflection of the academic caliber of research in this domain. The search strategy employed in this study was as follows: “[((TS = (refractory angina OR refractory angina pectoris OR refractory coronary artery disease OR refractory unstable angina pectoris)) AND LA = (English))] AND PY = (2003–2022).” Applying this strategy yielded a total of 1,723 results within the SCI-E database. To ensure the data's relevance and reliability, unqualified document types were excluded, and only articles and reviews were selected, resulting in a final set of 1,386 publications. These publications' complete records, encompassing titles, authors, abstracts, keywords, and cited references, were then exported from WOS and imported into CiteSpace 6.2.R2. This step served to eliminate any duplicate documents, ultimately leaving us with a collection of 1,386 unique publications, which formed the basis of this bibliometric research.

### 2.2. Methods

For the purpose of visualization and bibliometric analysis, CiteSpace was employed as a tool that focuses on critical points in academic research or the progression of a particular field or subject (5). After removing duplicates, all the records were imported into CiteSpace 6.2.R2. to facilitate further analysis. The parameter settings employed in this study were as follows: the time period considered spanned from January 2003 to December 2022, with a time slicing interval of one year. The selected node types for analysis included author, institution, country, keyword, reference, and cited author. To identify the most significant items within each time slice, the top 50 levels of the most cited or frequently occurring items were chosen based on the selection criteria. However, due to limitations in the network size of this particular version of CiteSpace software, which had a maximum threshold of 500, in cases where the network size exceeded this limit, the maximum value that the software could run of the top *n* items for each time slice was selected for subsequent analysis.

## 3. Results

### 3.1. Document characteristics

**Figure 1:**
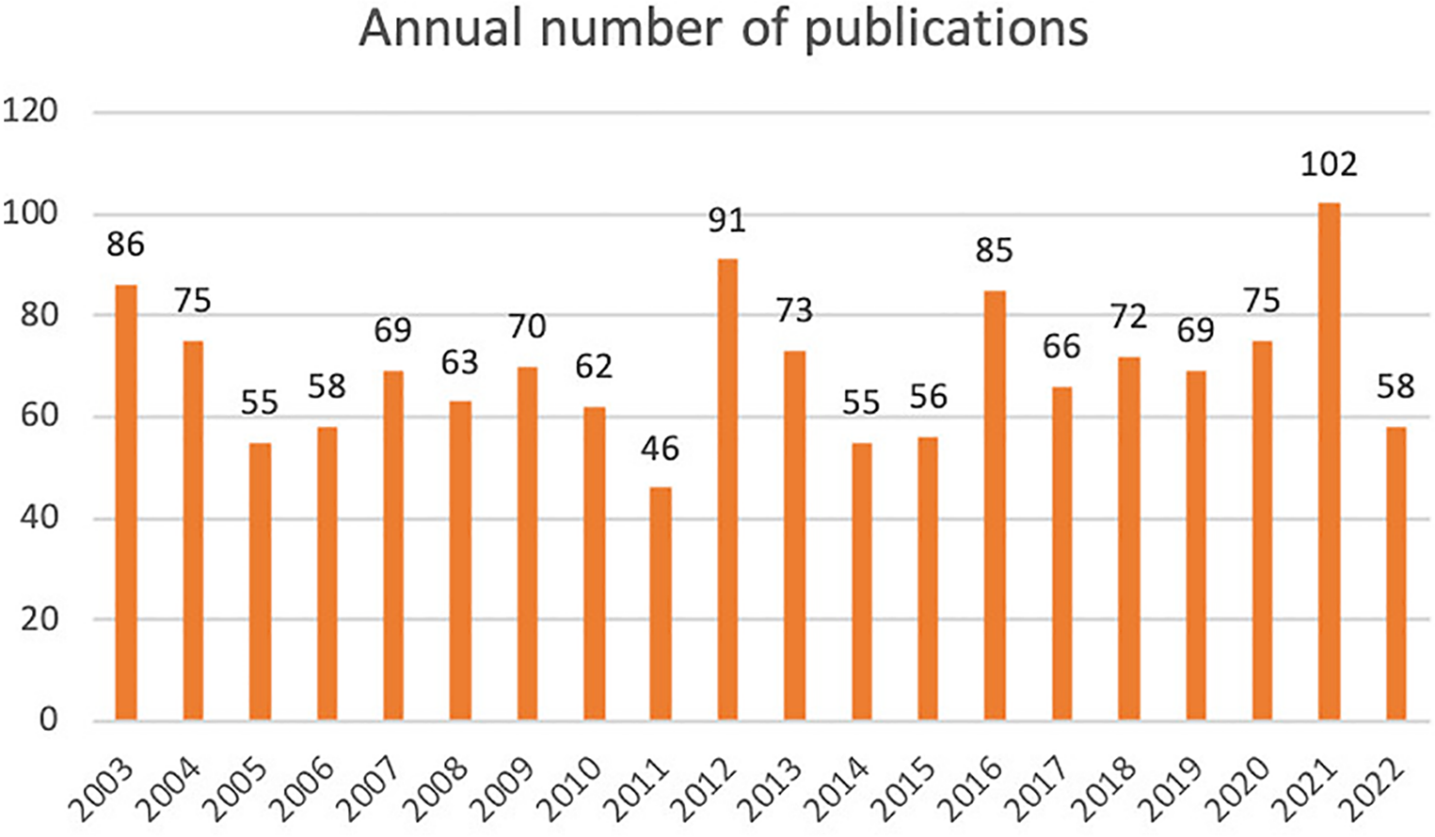
Annual number of publications about refractory angina.

Among the 1,386 carefully selected publications, 1,121 were identified as articles, while the remaining 265 were categorized as reviews. Throughout the span of the past 20 years, the average annual publication count for refractory angina stood at approximately 69. Figure 1 provides a visual representation of the annual publication trend in the field of refractory angina pectoris, showcasing a serrated pattern. Notably, there was a peak of 102 publications in 2021, contrasting with a low point of 46 publications in 2011. Additionally, minor publishing climaxes were observed roughly every four or five years. Furthermore, the number of articles surpassed that of reviews by a factor of more than four, signifying the substantial emphasis researchers place on experimental research within this field.

### 3.2. Cooperation Map of country/region and institute

**Figure 2:**
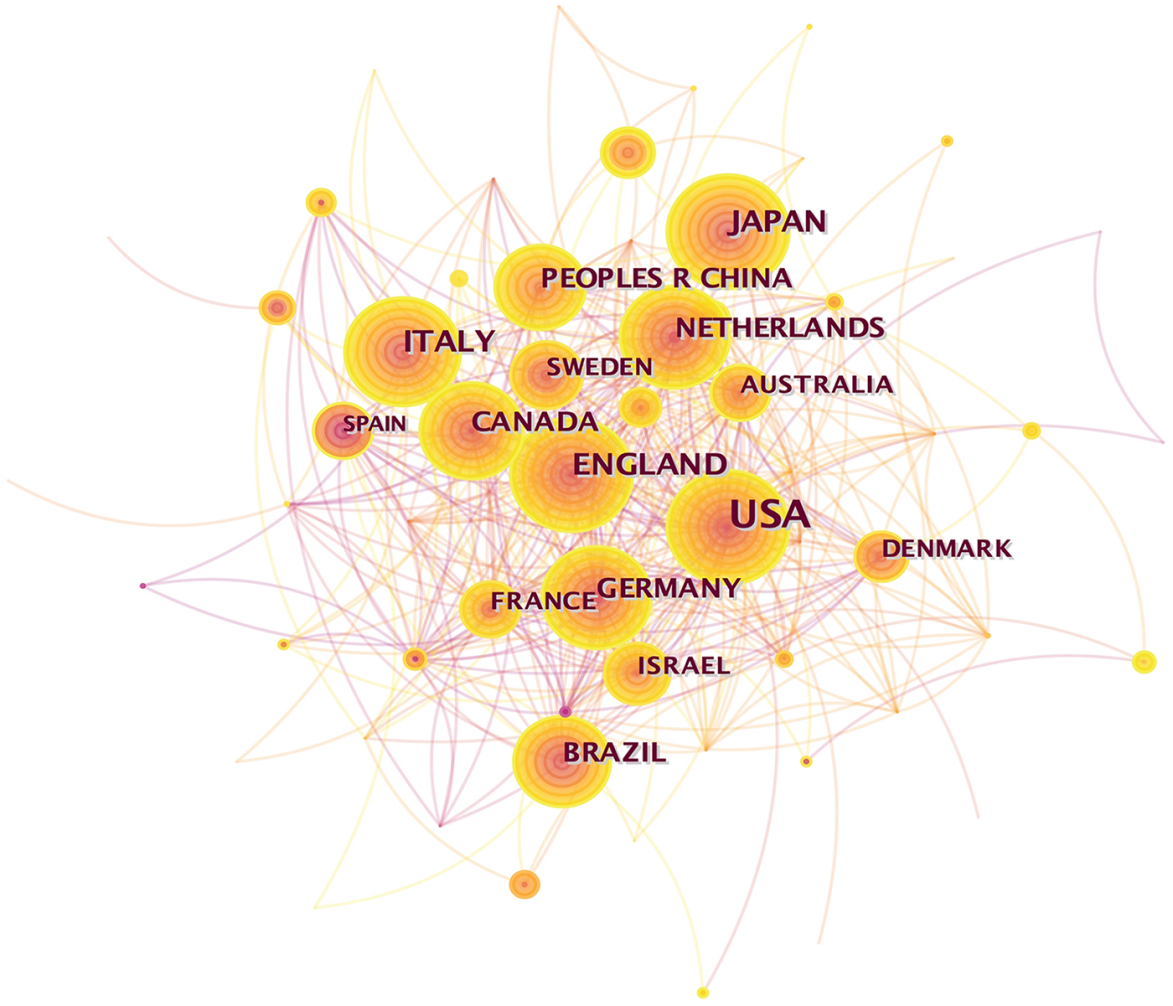
Country collaborative network analysis.

As is showed in Figure 2, we can see a complicated network with numerous lines, vividly illustrating the close collaboration between countries involved in refractory angina research. This network analysis encompassed 64 countries (*n* = 64), revealing 435 connections (*e* = 435) between them, corroborating the aforementioned observation. Notably, eight nations made substantial contributions with over 80 publications each. Leading the pack was the United States with an impressive 515 articles, nearly four times more than the second-ranked country. The top five countries in terms of publication volume were the United States (515), England (112), Italy (109), Japan (108), and Canada (86) (Table 1), underscoring the significant influence of the United States in the field of refractory angina. Furthermore, countries exhibiting a betweenness centrality (BC) value greater than 0.1 included the USA (0.62), England (0.13), and Germany (0.11). It is noteworthy that the USA, apart from its high publication volume, also possessed a substantial BC value, indicating its pivotal role in global research on refractory angina.

**Table 1:** Top 10 countries in terms of the number of refractory angina publications.

Rank	Country	Count	BC	Year
1	USA	515	0.62	2003
2	England	112	0.13	2003
3	Italy	109	0.07	2003
4	Japan	108	0.01	2003
5	Canada	86	0.09	2003
6	Germany	84	0.11	2003
7	Netherlands	84	0.08	2003
8	Peoples R China	82	0.03	2003
9	Brazil	57	0.04	2003
10	France	44	0.09	2003

BC, betweenness centrality.

**Figure 3:**
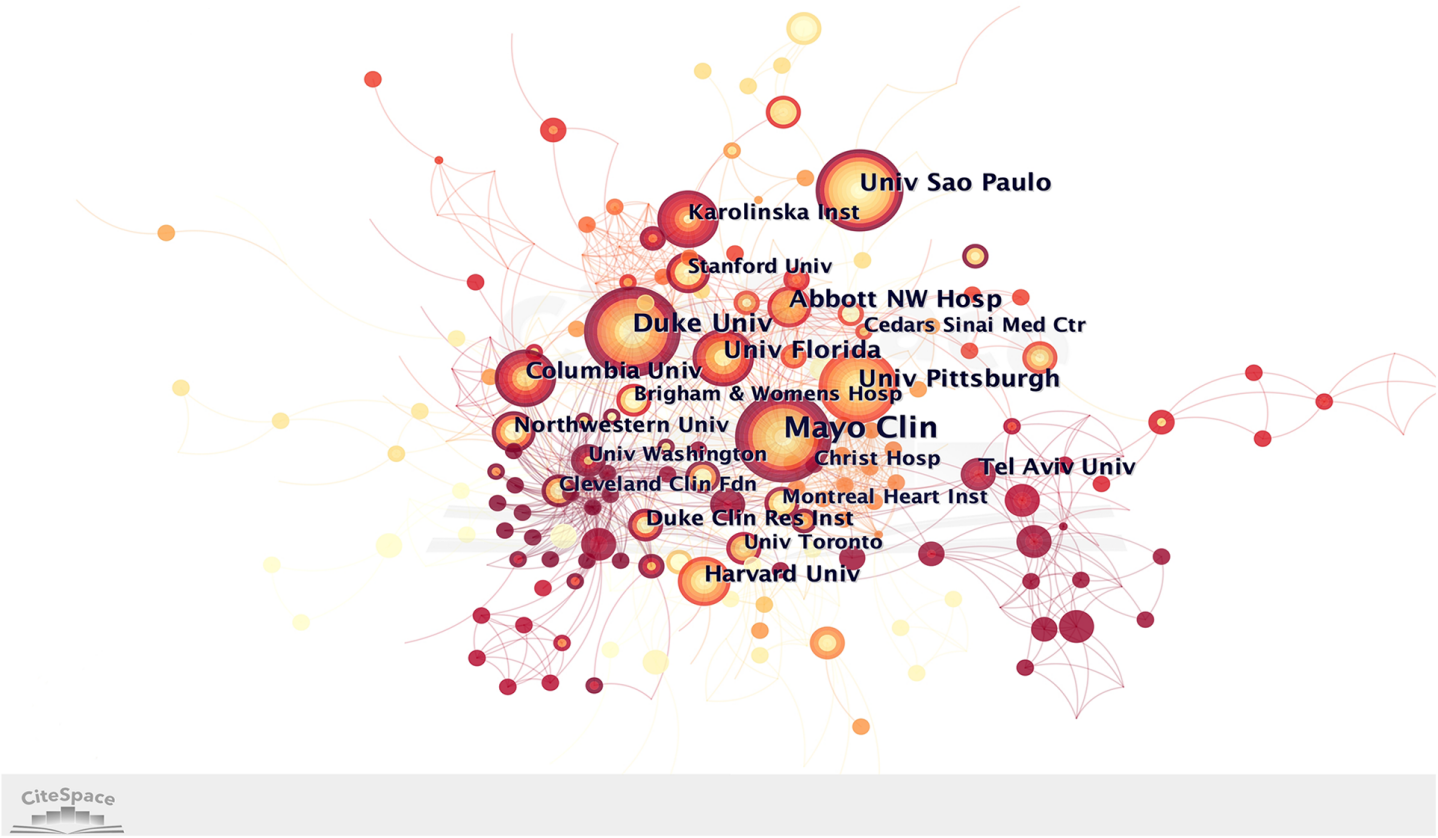
Institution collaborative network analysis.

Figure 3, displaying numerous connections among different institutions, showcases a lower density (0.0075) compared to Figure 2 (0.2158). This discrepancy suggests that the level of cooperation between institutions is not as extensive as that observed between countries. A total of 558 institutions worldwide were involved in refractory angina research. The top five institutions contributing to this field were Mayo Clinic (47), Duke University (27), University of Sao Paulo (22), University of Pittsburgh (20), and Abbott Northwestern Hospital (19) (Table 2). Interestingly, no institution possessed a BC value greater than 0.1, indicating that the extent of collaboration between institutions is relatively limited. However, it is worth highlighting that Tel Aviv University, emerging in 2018, published 15 articles and secured the eighth position among the top ten institutions. This observation suggests that Tel Aviv University has likely made noteworthy contributions to the field of refractory angina in recent years.

**Table 2:** Top 10 institutions in terms of the number of refractory angina publications.

Rank	Institution	Count	BC	Year	Country
1	Mayo Clin	47	0.07	2003	USA
2	Duke Univ	27	0.06	2003	USA
3	Univ Sao Paulo	22	0.01	2003	Brazil
4	Univ Pittsburgh	20	0.05	2003	USA
5	Abbott NW Hosp	19	0.03	2008	USA
6	Univ Florida	19	0.03	2004	USA
7	Columbia Univ	18	0.04	2005	USA
8	Tel Aviv Univ	15	0.04	2018	Israel
9	Harvard Univ	15	0.01	2004	USA
10	Karolinska Inst	14	0.02	2004	Sweden

### 3.3. Author and co-cited author

**Figure 4:**
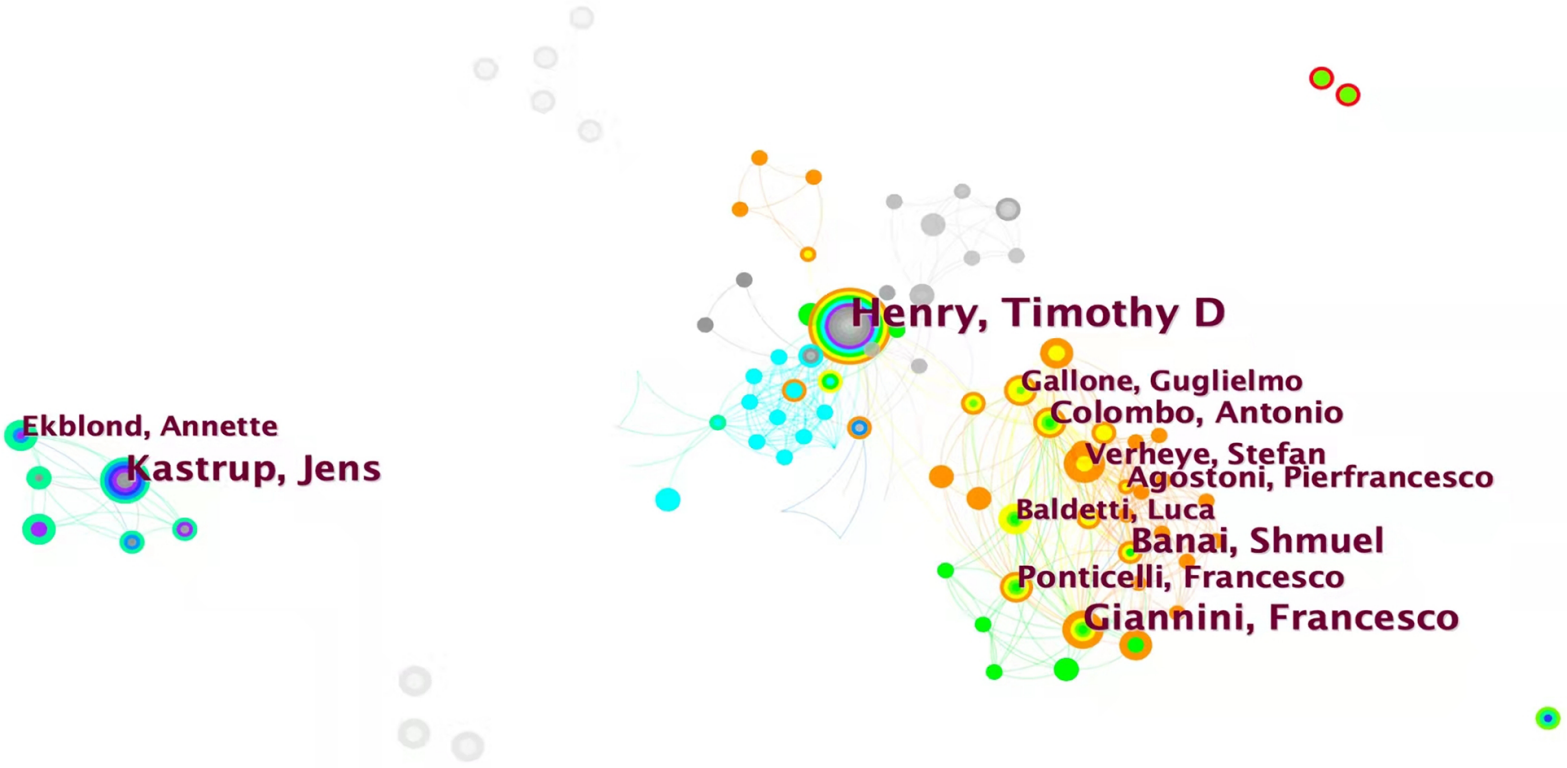
Author collaborative network analysis.

Within this bibliometric analysis, it becomes evident that only four authors have contributed more than 10 articles each. The top five authors with the highest publication counts are Henry Timothy D (26), Kastrup Jens (19), Giannini Francesco (16), Banai Shmuel (16), and Colombo Antonio (9). Notably, Giannini Francesco, who emerged in 2018, secured the third position on the list, showcasing significant contributions to the field of refractory angina, particularly regarding the coronary sinus reducer (6–8). Furthermore, when considering the BC values of the top ten authors, it becomes apparent that only two authors possess a BC value greater than 0, suggesting a relatively weak research cooperation relationship in the field of refractory angina (Figure 4, Table 3).

**Table 3:** Top 10 authors in terms of the number of refractory angina publications.

Rank	Author	Count	BC	Year
1	Henry, Timothy D	26	0.02	2007
2	Kastrup, Jens	19	0.00	2011
3	Giannini, Francesco	19	0.01	2018
4	Banai, Shmuel	16	0.00	2007
5	Colombo, Antonio	9	0.00	2018
6	Ekblond, Annette	9	0.00	2013
7	Verheye, Stefan	8	0.00	2020
8	Ponticelli, Francesco	8	0.00	2018
9	Agostoni, Pierfrancesco	8	0.00	2015
10	Konigstein, Maayan	8	0.00	2018

**Figure 5:**
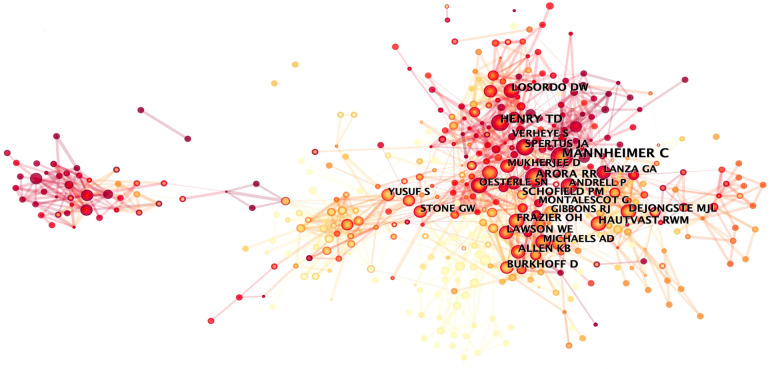
Author co-citation analysis.

Co-citation analysis identifies authors whose papers are simultaneously cited by a third author, establishing a co-citation relationship. Another point to note is that for co-citation analysis, CiteSpace software only extracts the lead author. It listed the top 10 authors in terms of co-citation frequency in Table 4, but failed to mention some authors with high BC values. Anteman EM (41, 0.29), Stone GW (65, 0.24), Spertus JA (83, 0.15), Boden WE (47, 0.15), and Leon MB (52, 0.13) are the top five among them, indicating that these authors are widely cited throughout the refractory angina study. It is worth noting that Henry TD appears at the forefront in both the authors and co-cited authors ranking, further emphasizing his significant contributions and strong influence in the field of refractory angina (Figure 5).

**Table 4:** Top 15 most cited authors of publications about refractory angina.

Rank	Cited Author	Count	BC	Year
1	Mannheimer C	213	0.04	2003
2	Henry TD	132	0.04	2003
3	Arora RR	112	0.05	2003
4	Losordo DW	87	0.05	2003
5	Frazier OH	87	0.03	2003
6	Allen KB	87	0.05	2003
7	Burkhoff D	84	0.01	2003
8	Spertus JA	83	0.15	2003
9	Schofield PM	78	0.07	2003
10	Dejongste MJL	75	0.02	2003

### 3.4. Keyword

Keywords serve as concise representations of an article's content, conveying its central ideas clearly and intuitively. The top 20 keywords were presented on co-occurrence frequency in refractory angina field in the Table 5. Among them, the occurrence frequency of Coronary article disease, Refractory angina, Spinal cord stimulation, Myocardial infiltration, Therapy, Refractory angina spectator occurred more than 100 times. Additionally, two keywords, “artery disease” and “refractory angina spectator” (BC = 0.14), whose BC value is greater than or equal to 0.1, were not listed in the table, playing an important role in connecting the entire refractory angina keyword network.

**Table 5:** Top 20 keywords of the publications of refractory angina.

Rank	Keywords	Count	BC
1	Coronary artery disease	318	0.02
2	Refractory angina	207	0.05
3	Spinal cord stimulation	139	0.03
4	Myocardial infarction	131	0.06
5	Therapy	108	0.07
6	Refractory angina pectoris	108	0.02
7	Management	97	0.07
8	Angina pectoris	95	0.05
9	Quality of life	86	0.09
10	Acute myocardial infarction	85	0.07
11	Disease	85	0.04
12	Heart failure	81	0.08
13	Double blind	79	0.04
14	Efficacy	73	0.06
15	Heart disease	67	0.05
16	Pectoris	63	0.02
17	Artery disease	60	0.10
18	Risk	60	0.07
19	Medical therapy	57	0.07
20	Kawasaki disease	56	0.04

**Figure 6:**
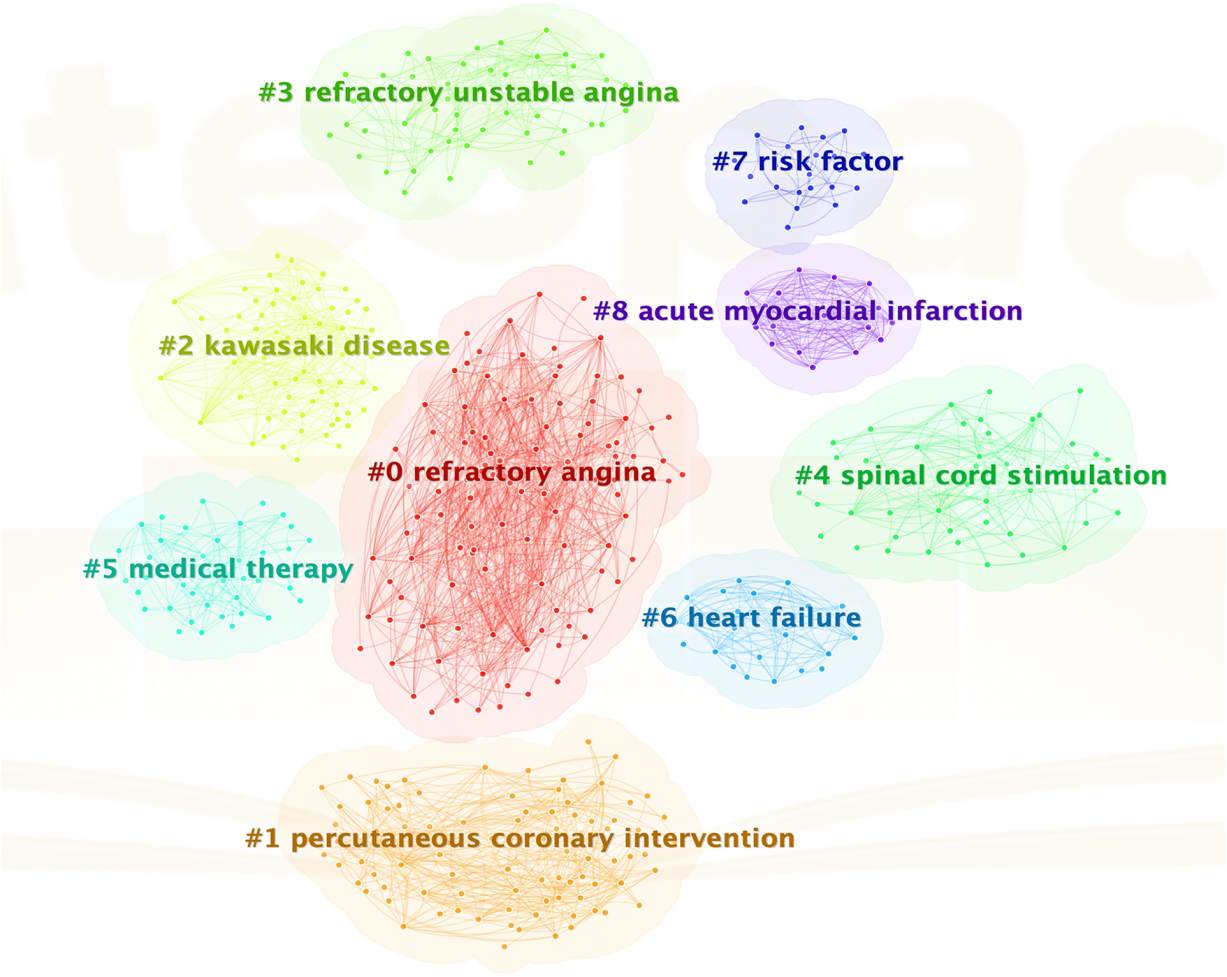
Keyword cluster analysis.

Cluster analysis is a statistical method that groups research objects into relatively homogeneous clusters based on their properties. CiteSpace software provides two indicators, modularity (abbreviated as *Q* value) and silhouette (abbreviated as S value), to evaluate the effectiveness and reliability of the cluster structure. A *Q* value greater than 0.3 indicates a significant cluster structure, while an *S* value greater than 0.7 suggests a convincing cluster. In this analysis, the *Q* value is 0.5197, and the *S* value is 0.7774, indicating a significant cluster structure and high reliability of the clustering relationships. As is displayed in Figure 6, there are nine labels by natural clustering, which are refinery angina (#0), recurrent coronary intervention (#1), Kawasaki disease (#2), refinery unstable angina (#3), spinal cord simulation (#4), medical therapy (#5), heart failure (#6), risk factor (#7), and acute myocardial infarction (#8). These cluster labels encompass aspects related to the disease, treatment, prognosis, and other relevant areas.

**Figure 7:**
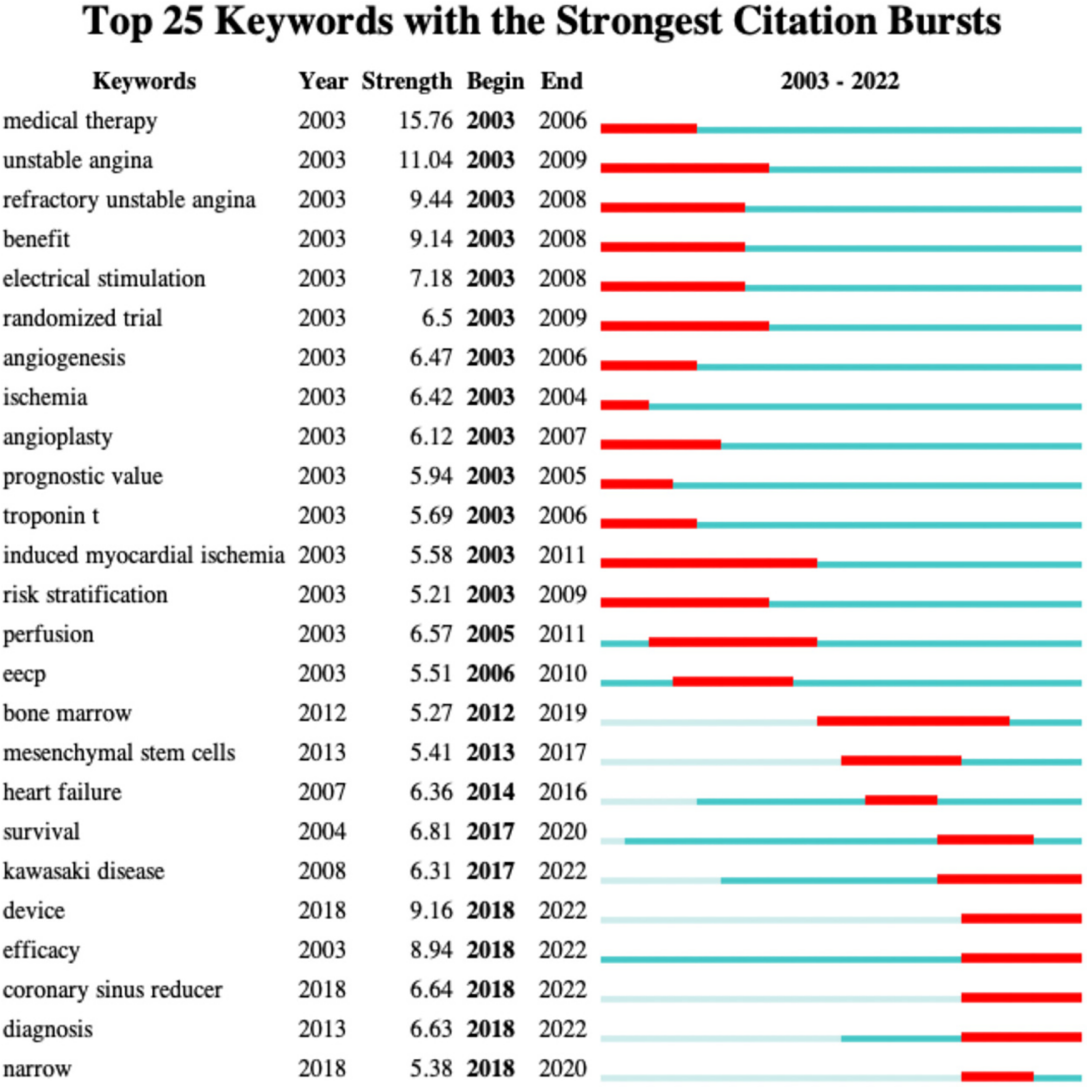
Top 25 keywords in burst analysis.

Burst term analysis in CiteSpace detects significant activity between nodes over a specific period, offering insights into research hotspots and their changes. As we can see in Figure 7, firstly, scientists focused more on unstable angina, refractory unstable angina, and in terms of diseases from 2003 to 2010. And as for the treatment of refractory angina, more efforts were made to seek solutions on medical therapy, angioplasty, electrical simulation, and enhanced external counterpulsation (EECP). Numerous clinical randomized trials were conducted during this period, utilizing performance and troponin T as evaluation indicators, while also examining patient prognosis, treatment benefits, and risk stratification. Secondly, during the five-year period from 2012 to 2017, heart failure became a more focused disease, with scientists focusing on stem cell therapy in treatments. Thirdly, in the past five years, from 2017 to the present, scientists have explored the relationship between Kawasaki disease and refractory angina. Coronary Sinus reducer has become a new hot topic in treatment, and scientists have once again focused on survival prognosis and treatment efficacy.

### 3.5. Reference co-citation analysis

**Figure 8:**
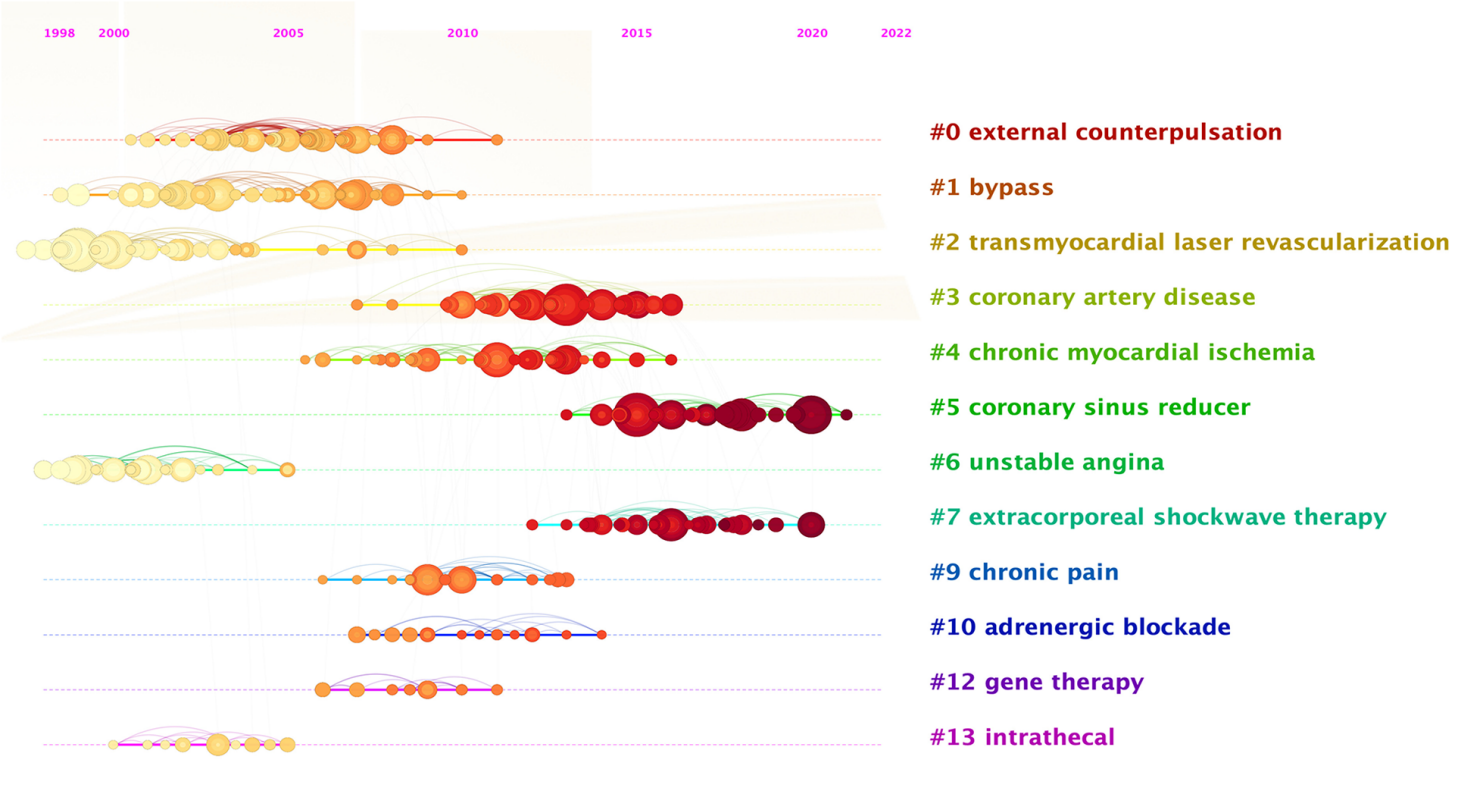
Timeline view of references.

The concept of co-citation, a research method for measuring the relationship between documents, was first introduced by American intelligence scientist H. G. Small in 1973. Co-citation refers to the situation where two or more papers are cited together in subsequent papers, indicating a connection between them. The top 20 most cited documents in the field of refractory angina were presented in Table 6, encompassing clinical trials, reviews, and practice guidelines. Among these, clinical trial articles emerged as the most frequently cited. And Figure 8 showed us the literature co citation network in this field. The highest cited article titled “Efficacy of a device to narrow the coronary sins in clinical angina,” authored by Stefan Verheye and published in The New England Journal of Medicine in 2015, garnered 44 citations and had a BC value of 0.14, highlighting its significant impact on the advancement of refractory angina research. The second most cited article was a practice guideline titled “2013 ESC guidelines on the management of stable coronary art disease: the Task Force on the management of stable coronary art disease of the European Society of Cardiology,” published in the European Heart Journal in 2013. This guideline held a BC value of 0.16, underscoring its crucial role in the field of refractory angina. Notably, the majority of highly cited articles consisted of clinical trial research papers investigating various treatment methods, suggesting that the treatment of refractory angina remains an active area of research with limited established guidelines. Furthermore, the timeline map of cited reference showed us the relationship between the cited literature, time, and topic in the form of keyword clustering, illustrating that the areas with more cited reference in recent years are coronary sinus reducer and extracorporeal shockwave therapy (Figure 8).

**Table 6:** Top 20 most cited references of publications about refractory angina.

Rank	Title	Count	BC	Reference type	Author	Journal	Year
1	Efficacy of a device to narrow the coronary sinus in refractory angina (9)	44	0.14	Article (clinical trial)	Veheye S	The new England journal of medicine	2015
2	2013 ESC guidelines on the management of stable coronary artery disease: the Task Force on the management of stable coronary artery disease of the European Society of Cardiology (1)	40	0.16	Article (practice guideline)	Montalescot G	European heart journal	2013
3	Transmyocardial revascularization with a carbon dioxide laser in patients with end-stage coronary artery disease (10)	39	0.02	Article (clinical trial)	Frazier OH	The new England journal of medicine	1999
4	Transmyocardial laser revascularisation in patients with refractory angina: a randomised controlled trial (11)	39	0.01	Article (clinical trial)	Schofield PM	Lancet	1999
5	Comparison of transmyocardial revascularization with medical therapy in patients with refractory angina (12)	37	0.00	Article (clinical trial)	Allen KB	The new England journal of medicine	1999
6	Diagnosis, Treatment, and Long-Term Management of Kawasaki Disease: A Scientific Statement for Health Professionals From the American Heart Association (13)	36	0.01	Review	McCrindle BW	Circulation	2017
7	Long-term survival in patients with refractory angina (14)	36	0.02	Article	Henry TD	European heart journal	2013
8	Transmyocardial laser revascularisation compared with continued medical therapy for treatment of refractory angina pectoris: a prospective randomised trial. ATLANTIC Investigators. Angina Treatments-Lasers and Normal Therapies in Comparison (15)	34	0.00	Article (clinical trial)	Burkhoff D	Lancet	1999
9	Transmyocardial revascularization with CO2 laser in patients with refractory angina pectoris. Clinical results from the Norwegian randomized trial (16)	30	0.07	Article (clinical trial)	Aaberge L	Journal of the American college of cardiology	2000
10	Intramyocardial, autologous CD34 + cell therapy for refractory angina (17)	29	0.01	Article (clinical trial)	Losordo DW	Circulation research	2011
11	2019 ESC Guidelines for the diagnosis and management of chronic coronary syndromes (18)	28	0.01	Article (practice guideline)	Knuuti J	European heart journal	2020
12	Treatment of refractory angina in patients not suitable for revascularization (19)	24	0.06	Review	Henry TD	Nature reviews. Cardiology	2014
13	ACC/AHA 2002 guideline update for the management of patients with chronic stable angina–summary article: a report of the American College of Cardiology/American Heart Association Task Force on Practice Guidelines (Committee on the Management of Patients With Chronic Stable Angina) (20)	24	0.04	Article (guideline)	Gibbons RJ	Circulation	2003
14	Thoracic spinal cord stimulation improves functional status and relieves symptoms in patients with refractory angina pectoris: the first placebo-controlled randomised study (21)	23	0.33	Article (randomized controlled trial)	Eddicks S	Heart (British cardiac society)	2007
15	The Reducer device in patients with angina pectoris: mechanisms, indications, and perspectives (22)	23	0.02	Review	Konigstein S	European heart journal	2018
16	The RENEW Trial: Efficacy and Safety of Intramyocardial Autologous CD34(+) Cell Administration in Patients With Refractory Angina (23)	23	0.02	Article (clinical trial)	Povsic TJ	JACC. Cardiovascular intervention	2016
17	Management of patients with refractory angina: Canadian Cardiovascular Society/Canadian Pain Society joint guidelines (24)	22	0.21	Article (practice guideline)	McGillion M	The Canadian journal of cardiology	2012
18	Spinal cord stimulation in the treatment of refractory angina: systematic review and meta-analysis of randomised controlled trials (25)	21	0.12	Review (meta-analysis)	Taylor RS	BMC cardiovascular disorders	2009
19	The problem of chronic refractory angina; report from the ESC Joint Study Group on the Treatment of Refractory Angina (26)	21	0.03	Review	Mannheimer C	European heart journal	2002
20	Safety and efficacy of a device to narrow the coronary sinus for the treatment of refractory angina: A single-centre real-world experience (27)	21	0.01	Article (A single-centre real-world experience)	Abawi M	Netherlands heart journal	2016

## 4. Discussion

This study employed CiteSpace software to conduct a comprehensive bibliometric analysis of publications in the field of refractory angina from 2003 to 2022. The analysis encompassed spatiotemporal distribution, author and institutional contributions, core literature, research hotspots, and trends in refractory angina research. The annual publication volume in this field demonstrated relative stability without significant growth, indicating that refractory angina has not experienced a sudden surge in popularity within cardiovascular disease research compared to other areas. The analysis of national cooperation networks revealed that the United States and European countries dominated research in the field of refractory angina, with limited participation from Asian and African countries. Japan and China were the sole Asian countries at the forefront of research. Similarly, top-ranked research institutions primarily consisted of universities and medical institutions from the United States and European countries, and there is barely collaboration between institutions. Author collaborative network analysis identified Henry TD as a pivotal researcher in the field of refractory angina, with a substantial number of publications and citations. Henry TD is renowned for his groundbreaking work in interventional cardiology and stem cell therapy in the United States. Another noteworthy author is Mannheimer C, who ranked first in terms of co-citation and was nearly twice as many as second place despite having fewer publications. Mannheimer C led the report on the treatment of refractory angina by the ESC Joint Study Group in 2002.

Keyword clustering and burst analysis revealed research trends and hotspots in refractory angina. Scientists focused on diseases related to refractory angina, such as unstable angina, myocardial ischemia, acute myocardial infarction, and heart failure. Recently, Kawasaki disease has gained attention as a prominent topic. Extensive clinical trials were conducted, and patient benefits, quality of life, and survival prognosis have always been emphasized in this field. The experimental hotspots and research hotspots in this field are both focused on the treatment of refractory angina. Scientists initially focused on medical therapy and angiogenesis. The ECS guidelines recommended the first-line treatment drugs as blocker and/or calcium channel blocker using alone or in combination to control angina, and long-acting nitrates, Ivabradine, Nicorandil, Ranolazine or Trimetazidine can be used as second-line personalized therapy (18, 28). However, clinical studies have failed to identify highly specific anti-angina drugs (29), and the effect of medical therapy for refractory angina is not ideal, though the medical therapy is an essential basic treatment for refractory angina. Angiogenesis, including PCI and plain old balloon angioplasty (POBA), exhibited positive effects in reducing angina frequency, improving quality of life and reducing the risk of adverse cardiovascular events (30–33). But patients with intractable angina pectoris may still experience angina pectoris symptoms after angiogenesis due to distal vascular disease, vasospasm, negative emotion, or other unknown reasons (28). Subsequently, scientists explored auxiliary treatment methods for refractory angina such as spinal cord stimulation and EECP. In 1987, Murphy et al. first reported that spinal cord stimulation can be used to treat refractory angina (34). In recent years, multiple clinical trials and large-scale meta-analysis have demonstrated significant therapeutic effects of spinal cord stimulation surgery. Patients treated with spinal cord stimulation surgery have a higher quality of life, and the long-term follow-up effect is significant (18, 35, 36). EECP is a non-invasive assistive circulatory device that helps increase cardiac blood flow, improve coronary artery supply, reduce cardiac ejection resistance, reduce myocardial oxygen consumption, and thus alleviate and eliminate symptoms of angina. EECP proved to be safe and effective in treating refractory angina through a large number of clinical trials (37–39). Besides, research has found that the relief rate of clinically refractory angina can reach as high as 72%, and more than half of RA patients have not experienced any recurrence of angina in this study (40). In recent years, stem cell therapy and coronary sinus reducer emerged as new treatment hotspots. CD34^+^ and CD133^+^ cell therapies are commonly seen in stem cell therapy. Although autologous stem cell therapy exhibited promise in reducing angina attacks and adverse cardiovascular events (17, 41), further investigation is necessary to ascertain its effectiveness for refractory angina (42). The coronary sinus reducer, initially proposed and applied to surgical procedures in the 1940s and 1950s (43, 44), then abandoned in the 1960s with the rise of bypass surgery, regained attention as a potential treatment option. Clinical trials confirmed its feasibility and safety (45–47), but additional research is needed to comprehend its mechanism of action, long-term benefits and individual adaptability (48).

Most of the treatment methods mentioned earlier, such as EECP and stem cell therapy, necessitate repetitive applications to maintain lasting effects, with coronary sinus reducer therapy being the only option that may exert permanent, rather than temporary, effects (49). Additionally, internal mammary artery occlusion therapy is currently garnering significant attention as a promising method with potential permanent effects (50). However, due to the limited number of literature on related topic words, this treatment method was not reflected in the data obtained in this study. Nonetheless, ample evidence suggests that the blood supply to the heart can be supplemented by non-coronary collateral circulation, which originates from surrounding arteries (51). One of the vital thoracic branches that establish connections with the heart is the pericardiophrenic branch of the internal mammary artery (52). The principle behind internal mammary artery occlusion is to ligate the internal mammary artery, creating a local hypertensive environment and increasing perfusion pressure in the channel leading to the heart (53). Meanwhile, angiogenic growth factors are applied to the internal mammary artery to enhance the development of new collateral branches, thereby promoting improved blood flow to the heart (54). Researches had shown that internal mammary artery occlusion can increase the blood supply of extracardiac ipsilateral coronary arteries, leading to reduced ischemia in dependent myocardial regions and alleviating angina symptoms in patients (55, 56). Although internal mammary artery occlusion has not yet gained widespread recognition as a standard treatment method and requires further evidence-based research, it is considered a novel approach for refractory angina or a potential alternative to coronary sinus reducer therapy due to its significant anti-myocardial ischemia effect (57). Overall, the treatment of refractory angina is still in the exploratory stage, and further studies are required to establish authoritative guidelines and evaluate long-term efficacy and cost-effectiveness.

Reference co-citation analysis identified five key publications that made significant contributions to the development of refractory angina. Eddicks in 2007 conducted the first placebo-controlled randomised study on thoracic spinal cord stimulation confirming that it can alleviate symptoms in patients with refractory angina (21). Taylor's meta-analysis in 2009 on spinal cord stimulation demonstrated its safety and efficacy as an alternative treatment method (25). McGillion, in 2012, led the Canadian Cardiovascular Society and the Canadian Pain Society in jointly publishing a practical guideline for the management of patients with refractory angina, provided evidence-based treatment recommendations for refractory angina (24). Subsequently, a guideline for the management of stable coronary artery disease published by the European Society of Cardiology in 2013, outlined treatment options for stable coronary artery disease and included recommendations for refractory angina (1). Lastly, Verheye's 2015 clinical trial on the coronary sinus reducer confirmed its effectiveness in improving symptoms and quality of life (9). These five articles serve as significant milestones in refractory angina research, especially three trial articles, aligning with recent research hotspots. However, the treatment of refractory angina necessitates further investigation, and the development of authoritative guidelines remains limited. High-quality randomized controlled trials, long-term evidence of benefits, and cost-effectiveness studies are crucial to advancing the field. This research aims to support clinical doctors and cardiovascular disease patients in enhancing their understanding of refractory angina.

## 5. Limitation

This study has several limitations. Firstly, the research content is limited to the analysis of literature published between 2003 and 2022, excluding earlier publications. Additionally, the analysis focused solely on English-language papers, potentially overlooking relevant research published in other languages. Furthermore, the study solely relied on the SCI-E database of WOS, which restricts the range of literature types considered. Secondly, the CiteSpace software utilized in the analysis has certain limitations, such as a network size limit of 500, which may impact the analysis results. Despite these limitations, this study offers a comprehensive analysis and summary of achievements in the field of refractory angina over the past two decades, contributing to scholars' understanding of its development status.

## 6. Prospect

Refractory angina, a condition characterized by persistent angina symptoms despite optimal medical therapy and revascularization, presents ongoing challenges in the field of cardiovascular medicine. While advancements have been made in understanding the underlying mechanisms and exploring potential treatment options, the outlook for refractory angina remains complex and multifaceted. In recent years, there have been notable developments in the field of refractory angina research. Scientists and clinicians have focused on expanding their knowledge of the disease, identifying novel therapeutic approaches, and evaluating their efficacy. This has led to the emergence of new treatment modalities and a better understanding of the disease process. One promising avenue of research lies in the exploration of alternative treatment methods beyond conventional medical therapy and revascularization such as spinal cord stimulation, EECP, and coronary sinus reducer, showing promise in the management of refractory angina. Advancements in regenerative medicine, particularly stem cell therapy, have also garnered attention in the field of refractory angina. In addition, some emerging methods for treating myocardial ischemia, such as internal mammary artery occlusion, can also try to treat refractory angina. Despite these advancements, it is important to acknowledge the remaining challenges in the management of refractory angina. The heterogeneity of the patient population and the complex underlying pathophysiology contribute to the variability in treatment response and outcomes. There is a need for further research to better understand patient selection criteria, optimal timing and sequencing of interventions, and long-term outcomes. Furthermore, the development of comprehensive and evidence-based guidelines for the management of refractory angina is crucial. Currently, treatment decisions are often based on expert opinion and individualized patient considerations. Establishing standardized guidelines will provide clinicians with a framework to navigate the complexities of treatment selection and optimize patient care.

## Data Availability

The original contributions presented in the study are included in the article/Supplementary Material, further inquiries can be directed to the corresponding author.
